# Editorial: Plant development and reproduction at single cell and cell type-specific resolution

**DOI:** 10.3389/fpls.2023.1261685

**Published:** 2023-08-07

**Authors:** Lynette R. Brownfield, Duarte D. Figueiredo, Michael Borg, Anja Schmidt

**Affiliations:** ^1^ Department of Biochemistry, University of Otago, Dunedin, New Zealand; ^2^ Max Planck Institute of Molecular Plant Physiology, Potsdam, Germany; ^3^ Max Planck Institute for Biology Tübingen, Tübingen, Germany; ^4^ Department of Plant Evolutionary Biology, Institute of Biology, University of Hohenheim, Stuttgart, Germany

**Keywords:** development, metabolomics, plant, reproduction, single cell, transcriptomics

Organogenesis and development of the plant body plan requires coordinated regulation of cell proliferation and fate specification to produce specialized cell types and cell lineages. Recent advances and method development in single cell or cell type-specific genomics and transcriptomics have provided a new toolbox to investigate plant development at single cell resolution (Ryu et al., 2021; Shaw et al., 2021; Cuperus, 2022). Such transcriptomic investigations provide unprecedented opportunities to systematically describe the genetic basis and regulatory programs governing development at high spatial and temporal resolution, which when combined with additional “omics” methods also help to place this in context of the specificities of cellular metabolism (Yu et al., 2023). Further, these techniques provide insights into the cellular and regulatory heterogeneity of developing and differentiated organs or tissues.

Methodological advances in these methods are continually providing an outlet to overcome the hurdles and challenges previously faced when profiling specific cell types. This includes the analysis of cell types that are difficult to access and that are in low abundance, such as cells of the developing male or female gametophytes within the floral tissues of higher plants (Schmidt et al., 2012; Schmid et al., 2015), or of cells embedded within seeds (Picard et al., 2021). Moreover, these advances allow developmental trajectories of given cells to be tracked over time and space, providing a fine resolution of developmental processes that would otherwise not be possible (Shahan et al., 2022; Nolan et al., 2023). Similarly, these technological improvements will allow transcriptional profiling of cells with high levels of secondary metabolites, from which isolation of high-quality RNA is usually problematic. Importantly, recent advances in state-of-the-art long read next generation sequencing has now opened doors to focus on non-model species. The scope of this Research Topic was to address technical approaches in transcriptomics and additional “omics” technologies and their application to different questions relating to cellular specification. Thereby the focus was not only on scientific findings but also on methodological improvements.

With this in mind, Lievre et al. present novel technical advances to analyze transcriptomes of pollen at distinct stages of development. The authors developed and established a new approach for transcriptional profiling of pollen from single anthers by conducting a chemical lysis that dissolves polysaccharides prior to RNA isolation with a subsequent enrichment of mRNAs. Importantly, as developmental stages are typically highly synchronized within the same anther, the approach allows for the precise staging and generation of biological replicates from a small number of plants. This enables comparative studies of defined developmental stages of wild-type or mutant lines as well as plants exposed to different stress conditions. While the established protocol was tested for the model species *Arabidopsis thaliana*, it was also shown to be effective in the horticultural crop kiwifruit (*Actinidia chinensis*), demonstrating its adaptation to non-model species.

The article from Tomoi et al. also highlights a technological improvement for studying the development of specific cell types and lineages. The authors describe genome editing of specific cells by optimization of the CRE/loxP system. Specificity and reproducibility of this protocol was enhanced by the use of a dual regulated system under the control of a newly-defined heat shock promoter in combination with CRE induction by dexamethasone. This technique can be used to generate chimeric tissues to study cellular development and communication with the support of transcriptomics and/or other “omics” approaches.

Analysis of biomolecules, particularly RNA, can be difficult in plant cells due to the high levels of secondary metabolites. Two articles in this Topic demonstrate the use of 10x Genomics technology to generate a transcriptome atlas from tissues containing secondary metabolites, including in non-model plants. The article of Song et al. describes the first single cell resolution transcriptome atlas for such a tissue type, namely in tuberous cassava roots (*Manihot esculenta*). The use of 10x Genomics technology enabled transcriptomes from almost 15,000 cells that were then grouped into 15 clusters based on orthologs of marker genes from model species. The study provides an important advance in the investigation of developmental trajectories in the sink organs of a staple food important to many tropical regions. In the second article, Zhou et al. used 10x Genomics technology to reveal differentiation trajectories of glandular trichomes in *Nepeta tenuifolia.* This species is used in traditional medical applications in Asia for its bioactive and volatile essential oils that are synthetized in its trichomes. Transcriptional profiles were generated for 33,254 protoplasts isolated from young leaves, which were partitioned into cell types with six broad populations and 19 clusters. The trichome cells were identified based on the expression of trichome-specific genes and the activity of genes involved in the synthesis of monoterpenes, which are an important component of the volatile oils.

Similarly, the article presented by Feng et al. is focused on a non-model species. Here, the advances of PacBio single-molecule real-time (SMRT) of RNA was used to investigate different tissues of *Nardostachys jatamansi*, an endangered species from the alpine Himalayas. In combination with gas chromatography-mass spectrometry (GC-MS) and ultrahigh performance liquid chromatography (UHPLC), the authors provide insights into the metabolism of volatile sesquiterpenoids and other aromatic compounds, which are of interest for medical purposes and drug preparation. In addition, a study by Deng et al. also applied SMRT technology to investigate other non-model species. Sequencing of seven distinct tissue types, including young and mature fruits, was used to generate a full-length transcriptome atlas of tree tomato or tamarillo (*Solanum betaceum* Cav.). This species is cultivated as a fast-growing fruit crop in several tropic and subtropic countries. Apart from the novel insights gained from this transcriptomic atlas, the data was further used to identify and develop simple sequence repeat (SSR) markers for phylogenetic analyses, which can be used for marker-assisted breeding purposes.

Taken together, this Research Topic comprises several articles presenting novel methodological advances in single cell and cell-type specific analysis. Although this technology is still in its infancy with significant limitations, including the loss of spatial information as well as the cost of entry, the future promises to provide researchers with unprecedented resolution in functional studies and plant breeding programs.

## Author contributions

LB: Conceptualization, Writing – review & editing. DF: Conceptualization, Writing – review & editing. MB: Conceptualization, Writing – review & editing. AS: Conceptualization, Writing – original draft.
